# IMPACT OF THE TEACHING NURSING HOME ON STAFF SATISFACTION AND BURNOUT

**DOI:** 10.1093/geroni/igad104.3415

**Published:** 2023-12-21

**Authors:** Howard Degenholtz, Jacqueline Dunbar-Jacob, Kim Ratliff, Donna Fick, Chelsea Dickson, Kenna Campbell, Erin Kitt-Lewis, Emily Franke

**Affiliations:** University of Pittsburgh, Pittsburgh, Pennsylvania, United States; University of Pittsburgh, Pittsburgh, Pennsylvania, United States; Wesley Enhanced Living, Media, Pennsylvania, United States; Penn State University, State College, Pennsylvania, United States; Jewish Healthcare Foundation, Pittsburgh, Pennsylvania, United States; University of Pittsburgh, Pittsburgh, Pennsylvania, United States; Penn State University, State College, Pennsylvania, United States; Jewish Healthcare Foundation, Pittsburgh, Pennsylvania, United States

## Abstract

The Teaching Nursing Home (TNH) is designed to improve the long-term care workforce and improve quality of care in nursing homes (NHs). The initiative is guided by an academic-practice partnership with mutual respect and shared learning and has multiple components: enhanced clinical rotations for nursing students with partner schools of nursing, implementation of the Institute for Healthcare Improvement Age-Friendly Health System “4M” quality improvement model, and an online learning network. Following the principles of participatory research, NH staff were engaged in the selection of outcomes and in decisions regarding data collection. The University of Pittsburgh collected staff satisfaction at two nursing homes, and burnout at three NHs. Baseline surveys were conducted in the winter of 2022 using a combination of web-based and in-person paper administration, and follow-up surveys were collected in 2023, one year after implementation. Response rates ranged from 14% to 54%. A total of 190 surveys were available at baseline, and 183 at follow-up. All surveys were anonymous; cross-sectional analyses were conducted to identify trends. Baseline burnout scores varied considerably across the three participating NHs and improved at two of the three NHs (p = .0000). By contrast, job satisfaction was unchanged in one NH and declined in one NH across two dimensions (p= .0058; p = .01216). After the first year of implementation, the TNH program is showing some benefit in terms of reducing burnout, a factor associated with turnover. As the Age-Friendly NH program is disseminated, it is important to track the impact on the workforce.

